# Fetal MRI: what’s new? A short review

**DOI:** 10.1186/s41747-023-00358-5

**Published:** 2023-08-10

**Authors:** Lucia Manganaro, Silvia Capuani, Marco Gennarini, Valentina Miceli, Roberta Ninkova, Ilaria Balba, Nicola Galea, Angelica Cupertino, Alessandra Maiuro, Giada Ercolani, Carlo Catalano

**Affiliations:** 1 Department of Radiological, Oncological and Pathological Sciences, Umberto I Hospital, Sapienza University of Rome, Rome, Italy; 2 National Research Council (CNR),, Institute for Complex Systems (ISC) c/o Physics Department Sapienza University of Rome, Rome, Italy; 3 Siemens Healthcare S.r.l., dARE, Milan, Italy

**Keywords:** Artifacts, Artificial intelligence, Diffusion magnetic resonance imaging, Fetus, Prenatal diagnosis

## Abstract

Fetal magnetic resonance imaging (fetal MRI) is usually performed as a second-level examination following routine ultrasound examination, generally exploiting morphological and diffusion MRI sequences. The objective of this review is to describe the novelties and new applications of fetal MRI, focusing on three main aspects: the new sequences with their applications, the transition from 1.5-T to 3-T magnetic field, and the new applications of artificial intelligence software. This review was carried out by consulting the MEDLINE references (PubMed) and including only peer-reviewed articles written in English. Among the most important novelties in fetal MRI, we find the intravoxel incoherent motion model which allow to discriminate the diffusion from the perfusion component in fetal and placenta tissues. The transition from 1.5-T to 3-T magnetic field allowed for higher quality images, thanks to the higher signal-to-noise ratio with a trade-off of more frequent artifacts. The application of motion-correction software makes it possible to overcome movement artifacts by obtaining higher quality images and to generate three-dimensional images useful in preoperative planning.

**Relevance statement**

This review shows the latest developments offered by fetal MRI focusing on new sequences, transition from 1.5-T to 3-T magnetic field and the emerging role of AI software that are paving the way for new diagnostic strategies.

**Key points**

• Fetal magnetic resonance imaging (MRI) is a second-line imaging after ultrasound.

• Diffusion-weighted imaging and intravoxel incoherent motion sequences provide quantitative biomarkers on fetal microstructure and perfusion.

• 3-T MRI improves the detection of cerebral malformations.

• 3-T MRI is useful for both body and nervous system indications.

• Automatic MRI motion tracking overcomes fetal movement artifacts and improve fetal imaging.

**Graphical Abstract:**

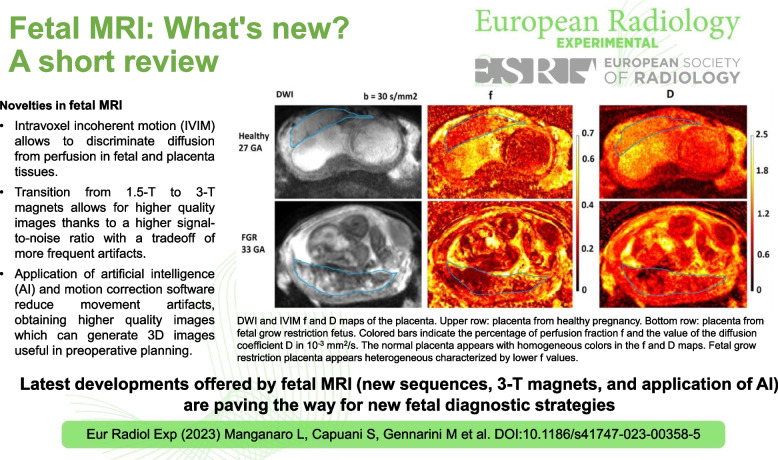

## Background

Fetal magnetic resonance imaging (MRI) is a complementary second-line imaging study usually performed after the routinely obtained anatomic ultrasonography (US), which provides important information in the evaluation of the fetus and the placenta [1, 2]. It has numerous advantages including safety, high resolution of tissue contrast, and it can overcome the limits of ultrasound examination. Indications for fetal MRI are for both the central nervous system and the fetal body.

Regarding central nervous system, the most frequent indications are ventriculomegaly, midline malformation, posterior fossa malformation, supratentorial parenchymal lesion or destruction, ischemic hemorrhagic lesions, infective disease, facial malformations (*e.g.,* cleft lip/palate), and neural tube defects [3].

In the evaluation of fetal body, generally, the indications to perform fetal MRI are congenital diaphragmatic hernia, neck masses, fetal airway obstruction and congenital pulmonary malformations, meconium peritonitis, sacrococcygeal teratoma, abdominal/pelvic masses, and lower urinary tract obstruction [4].

Recent technological advances have led to the development of new sequences and new software capable of providing and interpreting qualitative and quantitative data useful for investigating different pathological conditions and fetal malformations [5]. Furthermore, new studies reassure the safety of exposure of fetuses to high magnetic fields such as those from 3 T [6].

This review aims to describe the latest news in fetal MRI. In particular, the emphasis has been placed on the new fetal MRI sequences, with particular attention to diffusion-weighted imaging (DWI) techniques, including intravoxel incoherent motion (IVIM), on the transition from 1.5-T to 3-T magnets, and finally on the possibilities offered by the artificial intelligence (AI) and radiomics applied to fetal MRI.

## Literature search

The literature review was conducted by three investigators using search terms related to “fetus” and “MRI” by consulting the MEDLINE references (PubMed). The literature search was first performed from November 1, 2022, to December 20, 2022, and an updated repeat search of the literature was again performed from March 10, 2023, to March 31, 2023, using the identical search term plus “AI” and “radiomics.”

Only articles published from 2005 onwards were taken into consideration. No limits have been set as regards the number of studies to be taken into consideration. The inclusion criteria were for English-language peer-reviewed articles only. Case reports and opinion pieces were excluded.

Having covered a very heterogeneous number of publications, we did not have any predefined tools for bias assessment or quality appraisal, and it was not in our interest to exclude any studies on these grounds during the scoping phase. It was also considered inappropriate from the outset to carry out a statistical analysis of the literature. For this reason, it was decided to present the review of our results in a descriptive manner.

## DWI and IVIM

In recent years, fetal MRI has greatly increased our knowledge and diagnostic reliability for the evaluation of placental and fetal anomalies [7]. Thanks to DWI techniques [8], apparent diffusion coefficient (ADC) maps [9, 10], and IVIM [7–13] protocols, it is possible to obtain quantitative values for fetal microstructure and perfusion estimation that provide complementary information to US examinations. This is of crucial importance for prenatal counseling and risk stratification approach. Actually, the role of DWI is well known in several condition as ischemic and hemorrhagic lesion or in the brain, renal, or lung development process (Table 1) [14–16].

**Table 1:** Diffusion-weighted imaging (DWI) and intravoxel incoherent motion (IVIM) fetal studies

	**First author**	**Year**	**Title**	**Number of cases**	**Results**
1	Ercolani G	2021	Intravoxel incoherent motion (IVIM) MRI of fetal lung and kidney: can the perfusion fraction be a marker of normal pulmonary and renal maturation?	34	The IVIM perfusion fraction f may be considered as a potential marker of pulmonary and renal maturation in relation to hemodynamic changes described in intrauterine life
2	Jakab A	2018	Microvascular perfusion of the placenta, developing fetal liver, and lungs assessed with intravoxel incoherent motion imaging	33	Gestational age-associated changes of the placental, liver, and lung IVIM parameters likely reflect changes in placental and fetal circulation and characterize the trajectory of microstructural and functional maturation of the fetal vasculature
3	Antonelli A	2022	Human placental microperfusion and microstructural assessment by intra-voxel incoherent motion MRI for discriminating intrauterine growth restriction: a pilot study	67	Perfusion IVIM parameters velocity of ballistic flow and D* may be useful to discriminate different micro-vascularization patterns in intrauterine growth retardation being helpful to detect microvascular subtle impairment even in fetuses without any sign of ultrasound Doppler impairment in utero. Moreover, velocity of ballistic flow may predict fetuses’ body weight in intrauterine growth restriction pregnancies
4	Yuan X	2019	Fetal brain development at 25−39-week gestational age: a preliminary study using intravoxel incoherent motion diffusion-weighted imaging	79	IVIM DWI parameters may indicate fetal brain developmental alterations, but the conclusion is far from reached due to the not as high-powered correlation between IVIM parameters and GA
5	Di Trani M. G	2019	Apparent diffusion coefficient assessment of brain development in normal fetuses and ventriculomegaly	58	Apparent diffusion coefficient can be a useful biomarker of brain abnormalities associated with ventriculomegaly
6	Jiang L	2021	Probing the ballistic microcirculation in placenta using flow-compensated and non-compensated intravoxel incoherent motion imaging	40	The study shows the feasibility of using flow-compensated and noncompensated IVIM to noninvasively measure ballistic flow velocity in the placenta, which may be used as a new marker to evaluate placenta microcirculation
7	Liao Y	2022	Detecting abnormal placental microvascular flow in maternal and fetal diseases based on flow-compensated and non-compensated intravoxel incoherent motion imaging	61	Velocity of ballistic microcirculatory flow showed superior performance in the diagnosis of gestational diabetes mellitus and fetal growth restriction, indicating the potential of the joint flow-compensated and noncompensated IVIM method for placenta examinations

In this regard, a recent paper [17] highlights that perfusion IVIM parameters f and D* may be useful to discriminate different microvascularization patterns in small for gestational age (SGA) and fetal growth restriction (FGR) placentae. IVIM provides the concurrent estimation of the pure molecular water diffusion in extracellular space and microcirculation of blood water in randomly oriented capillaries (perfusion). Fetal FGR can be defined as an estimated fetal weight less than the 10th percentile for gestational age (GA) by prenatal ultrasound evaluation. The condition is associated with several short-term and long-term complications that can severely impact the quality of life. Even though SGA fetuses were generally considered “constitutionally small,” both SGA and FGR fetuses can have a low perinatal outcome and a certain form of placental dysfunction, indicating that SGA is more likely a group of a milder form of FGR than just a group of physiological “smaller” fetuses.

**Fig. 1:**
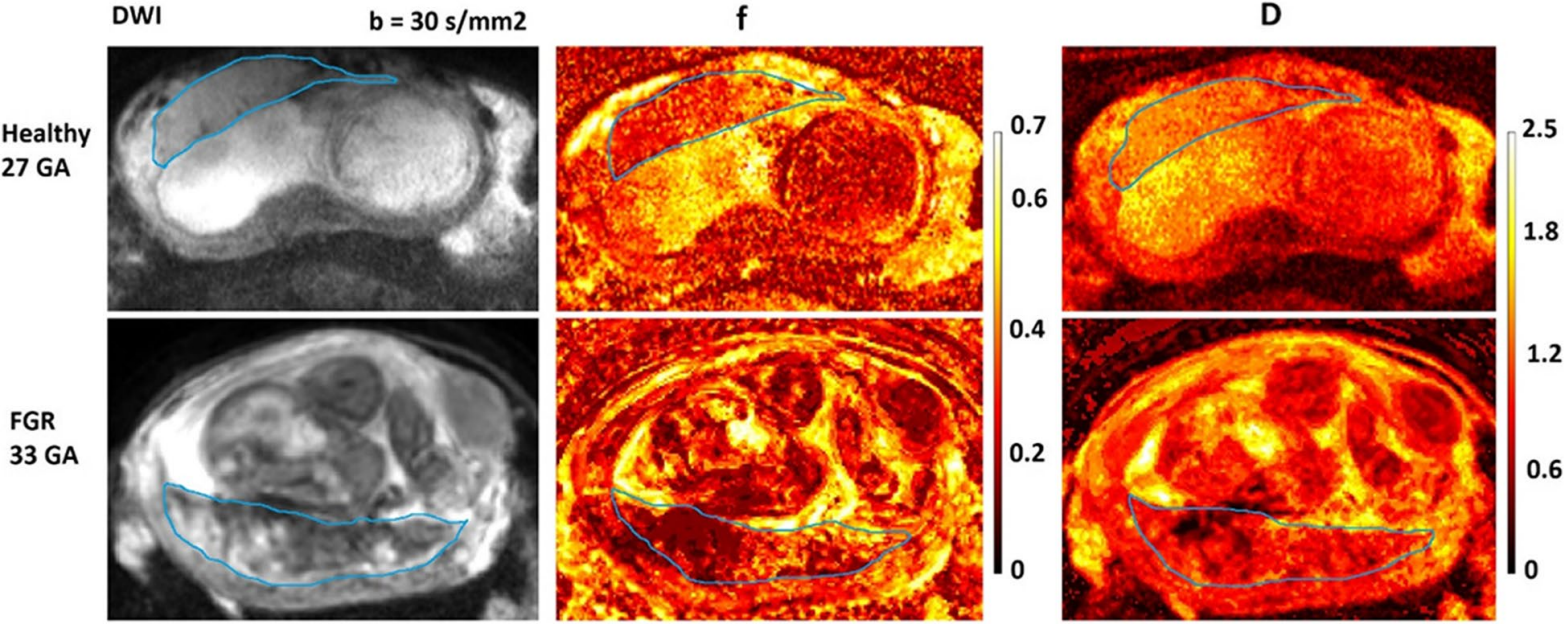
Diffusion-weighted imaging (DWI), obtained with *b* = 30 s/mm^2^, and intravoxel incoherent motion f and D maps of the placenta which is bordered by a thin blue line. Upper row: placenta from a woman with a normal 27-week healthy pregnancy. Bottom row: placenta from a female with fetal grow restriction fetus 33 weeks old. The colored bars indicate the percentage of perfusion fraction f and the value of the diffusion coefficient D in 10^−3^ mm^2^/s. The normal pregnancy placenta appears with homogeneous colors in the f and D maps. In contrast, the fetal grow restriction placenta appears heterogeneous characterized by lower *f*-values compared to the healthy placenta

In prenatal clinical practice, the assessment of different perinatal prognoses between SGA and FGR is crucial for properly managing the pregnancy. US examination alone does not discriminate SGA and FGR placentae because it does not allow for measuring perfusion in-depth and microvascularization. Conversely, in the placental tissue, the perfusion fraction parameter f quantifies the perfusion fraction of water molecules perfused in microcapillaries with a D* rate, and D* quantifies a sort of perfusion [11]. IVIM shows a significantly higher perfusion fraction f in normal placentas, lower in FGR ones (Fig. 1), and f assumes an intermediate value in SGA placentas [17]. Moreover, f measured in the placenta can predict body weight. Indeed, a significant positive correlation was found between f and body weight in FGR and SGA placentae, and a significant negative correlation was found between D and gestational age (GA) in SGA and FGR group but not in the normal pregnancy group [17]. Placental parenchyma in both SGA and FGR is characterized by various degrees of structural abnormalities with tissue heterogeneity, hypercellularity, and elevated deposition of fibrin by fibroblasts. These abnormalities may be responsible for the decrease in diffusion with the progress of gestation.

The key advantage of IVIM diffusion investigation is that it is a perfusion imaging modality not needing intravenous contrast medium administration. In the study of placental tissue (which is highly perfused), it is very important to obtain a concurrent estimation of perfusion and diffusion separately. Another useful application of IVIM concerns the investigation of the fetal body. In a recent paper [18], the IVIM perfusion fraction f shows the potential to monitor pulmonary and renal maturation concerning hemodynamic changes occurring in intrauterine life. Other authors [19] showed that the f of the liver decreased sharply during gestation, and lung maturation was characterized by increasing perfusion fraction.

With IVIM, it is possible not only to quantify the perfusion fraction but also to quantify the diffusion coefficient D without the bias due to the perfusion component present in the DWI signal obtained at low *b*-values (about less than 150 s/mm^2^). For this reason, the D parameter evaluated with the IVIM model is more sensitive to variations in tissue hypercellularity than the ADC parameter. This is also valid for the study of the fetal brain, much more perfused than the adult brain. However, to obtain a spatial resolution sufficient to investigate the fetal brain, the acquisition times of the IVIM protocol [20] could be too long. To overcome this drawback, but renouncing the perfusion estimate, fetal brains can be investigated by quantifying ADC. By normalizing DWI at different *b*-values data with the DWI at a *b*-value of 50 s/mm^2^, the perfusion bias in estimating the ADC value is strongly reduced, as shown in a recent paper [21]: ADC in centrum semiovale and frontal white matter significantly discriminated between normal fetal brain and ventriculomegaly.

Some authors have begun to propose variants of the IVIM model to make it more useful for fetal diagnostics. The joint flow-compensated and non-compensated IVIM model has been proposed to detect potential alterations of placental flows in FGR fetuses [22, 23]. The flow-compensated and non-compensated IVIM parameter velocity of ballistic flow showed superior performance in the diagnosis of FGR, compared to IVIM parameter [23].

## Fetal MRI at 3 T

In the last decades, 3-T scanners have increasingly progressed from research to clinical tools for fetal MRI (Table 2) [24]. The gain in signal-to-noise ratio from the higher field strength can be exploited to speed up the acquisition, necessary to overcome sudden fetal movement or improve spatial resolution for a more precise fetal depiction. Victoria et al. [25] compared fetal imaging on 1.5-T and 3-T MRI of fetuses of matched GA and similar anomalies, scoring the different images. Anatomical structures analyzed included the intestine, liver, kidney, airway, cartilage, and spine. The image evaluation score ranged from 0 to 4, with 4 being the best image quality. The study highlighted a general advantage of 3-T imaging, which obtained higher scores than 1.5-T imaging, the best scores being obtained using balanced steady-state-free precession (bSSFP) sequences [25].

**Table 2:** Fetal 3-T magnetic resonance imaging (MRI) studies

	**First author**	**Year**	**Title**	**Number of cases**	**Results**
1	Victoria T	2016	Comparison between 1.5-T and 3-T MRI for fetal imaging: is there an advantage to imaging with a higher field strength?	116	An overall advantage to performing fetal imaging at 3 T was made evident by the higher imaging scores obtained with 3-T MRI *versus* 1.5-T MRI when different fetal anatomic structures were evaluated
2	Bourgioti C	2022	Comparison between 1.5-T and 3.0-T MRI for the diagnosis of placenta accreta spectrum disorders	182	3.0-T MRI and 1.5-T MRI are equivalent for the diagnosis of placenta accreta spectrum
3	Colleran G. C	2022	Fetal magnetic resonance imaging at 3 Tesla — the European experience	27	The use of 3-T magnets in fetal MRI has improved the availability and quality of advanced imaging sequences and allowed for better anatomical evaluation. There remain significant challenges to minimize the impact of artifacts on image quality
4	Righini A	2022	Abbreviated turbo spin echo T2- and FLAIR-weighted sequences to complement multiplanar HASTE images in “Quick MRI” pediatric brain imaging at 3 Tesla: a child-tailored approach	N/A	The use of abbreviated versions of turbo spin-echo sequences is feasible in uncooperative children or those under 6 years of age. It could provide some advantages in diagnostic pediatric work-up and complement half-Fourier acquisition single-shot turbo spin-echo (HASTE) techniques in current “Quick MRI” imaging. In conditions such as neuro oncology, complex brain malformations or epilepsy such an approach is not suitable
5	Yadav B. K	2019	Quantitative susceptibility mapping in the human fetus to measure blood oxygenation in the superior sagittal sinus	21	This *in vivo* study demonstrates the feasibility of using quantitative susceptibility mapping in the human fetal brain to estimate magnetic susceptibility and venous oxygen saturation

As far as the study of the placenta is concerned, the recent study conducted by Bourgioti et al. [26] including 182 pregnant women showed that the diagnostic power of 1.5-T and 3-T MRI in the evaluation of the placenta accreta spectrum is substantially equivalent [26].

The first survey of European fetal imaging practice published in 2020 revealed that only 30% of fetal MRI are performed at 3 T. Among the reasons why the adoption of the 3-T MRI is still a minority, we find that not all centers have those magnets, the concern for possible damage caused to the fetus, and the increase in artifacts. Concerns regarding possible damage to the fetus were related to the increase in the specific absorption rate (SAR), the high noise generated by 3-T magnets with consequent possible impacts on the fetal auditory system, and finally the possibility of causing intrauterine growth retardation [27]. Despite these concerns, to date, there is no evidence that 3-T MRI causes growth retardation or damage to the auditory system [6, 28].

As the intensity of the magnetic field increases, the release of radiofrequency energy also increases, thus determining an increase in the SAR, whose limit for the maternal whole body has been set by the Food and Drug Administration in the USA at the threshold value of 4 W/kg [29]. For this reason, when switching from a 1.5-T magnet to a 3-T one, it is important to adjust the sequence parameters considering that safety systems prohibit exposure exceeding these threshold values [30].

The increase in field strength also causes artifacts such as chemical shift, *B*_1_ inhomogeneity, and standing wave [31]. Although these issues are present at 1.5 T as well, they become a clear problem to be addressed at 3 T. Dielectric artifacts can be challenging in fetal imaging. These appear as darker regions within the image generated by the radiofrequency waves which have shorter wavelengths than the object to be scanned [32]. This dielectric effect is emphasized if the patient abdominal diameters exceed the radiofrequency wavelength. This occurs in many pregnant women, especially because of the dielectric properties of the amniotic fluid [33]. In terms of image contrast, the relaxation times T1 and T2* differ between the respective tissue, depending on the magnetic field strength [31], and how to achieve the desired contrast is a challenge to face. In particular, the T2* of the fetal placenta is much lower at 3-T than at 1.5-T. This could compromise the correct evaluation of D and D* IVIM parameters. To overcome this drawback, DWI acquisitions should be performed with echo times largely shorter than T2*.

The recommendations of Fetal Imaging Task Force of the European Society of Pediatric Radiology [27] regarding preferred magnetic fields to be used for different indications in fetal MRI are as follows:

1. If available, it is preferable to use 3-T MRI for neurological indications, especially those involving the posterior cranial fossa, lesions of the brain parenchyma, and small median structures.
2. When available, 3-T MRI is preferred for indications of the fetal body, especially to recognize herniated organs in cases of diaphragmatic hernia, to evaluate smaller abdominal and pelvic organs, and to evaluate cartilages.
3. 1.5-T MRI is preferable in cases of polyhydramnios, due to the more pronounced shielding effects and resultant artifacts at 3 T.
4. GA should not affect the choice of magnetic field to use.

The International Society of Ultrasound in Obstetrics and Gynecology guidelines updated to 2023 [34] do not indicate a preference for 3 T or 1.5 T, recognizing on the one hand the ability of 1.5-T scanners to obtain good image resolution even at 18 months of GA and on the other hand the higher resolution of the image obtained by 3-T scanners at a comparable rate of energy deposition on tissues. However, for conditions such as polyhydramnios, they suggest using a 1.5-T scanner as it is less sensitive to fluid-wave-related artifacts [34].

## Fetal neuroimaging at 3 T

Studying the brain in small fetuses is one of the most challenging tasks for clinical imaging due to the unpredictable movements of the unborn child. Depicting small anatomical structures and differentiating the transient layers of the developing cerebral hemispheres, despite the expected low tissue contrast, pose critical challenges [35]. However, 3-T MRI scanners can help overcome these challenges in terms of contrast, thanks to some parameter corrections we have outlined in previous paragraphs. T2-weighted images provide the best contrast for highlighting the anatomical differences in the fetal brain, specifically the water and lipid content in the mature brain. Additionally, the high signal gained with stronger gradients can be exploited for higher resolution and acceleration factors.

The rapid imaging strategy employed in fetal MRI is a salutary practice that has already found utility in pediatric imaging. Turbo spin-echo sequences are particularly susceptible to motion artifact, which can compromise the accurate diagnosis of brain abnormalities. To address this challenge, Righini et al. [36] investigated a shorter two-dimensional (2D) turbo spin-echo and fluid-attenuated inversion-recovery (FLAIR) sequence for studying pediatric brains, capitalizing on the advantages of 3-T scanners. The abbreviated protocol with a duration of 52 s presents an intriguing option to be used in fetal brain, to determine whether the motion artifact is acceptable or should be excluded.

It is widely acknowledged that 3-T scanners offer significant advantages in the realm of neuroimaging, particularly with respect to fetal MRI, where the evaluation of fetal cerebral blood oxygenation status is a critical factor in identifying fetuses at risk of brain injury [37]. Crucial to this endeavor is the accurate susceptibility quantification of fetal cerebral vessels, a task that is achieved through quantitative susceptibility mapping (QSM) facilitated by susceptibility-weighted imaging (SWI), a multi-echo gradient sequence that is both lengthy and motion sensitive. However, emerging high acceleration imaging technologies offer a promising avenue for achieving optimal results with reduced scan time.

One such technology is wave-controlled aliasing in parallel imaging (CAIPI) SWI, which has been demonstrated to reduce brain scan time from 5 to 2 min in adults [38]. This technique is a fusion of two imaging techniques (Wave and CAIPI), which combine oscillated gradients applied in the slice and phase encoding direction during readout, resulting in a corkscrew *k*-space trajectory. Moreover, parallel imaging with a coil sensitivity knowledge reduces undersampling artifacts and g-factor noise, improving tolerance for high acceleration factors [39]. This technology represents an interesting perspective for fetal MRI, but its application still needs to be explored. The utility of Wave-CAIPI SWI is not limited to susceptibility quantification for QSM, as it is also applicable to other neuro sequences, such as T1-weighted “magnetization prepared rapid gradient-echo,” MPRAGE, T2-weighted “sampling perfection with application-optimized contrasts using different flip angle evolution,” and SPACE, for depicting even smaller structures within a reasonable acquisition time [40].

## Our 3-T MRI protocol

The International Society of Ultrasound in Obstetrics and Gynecology [34] guidelines suggest including at least T2-weighted sequences in three orthogonal planes of the fetal brain and body and T1-weighted and gradient-echo (GRE)-echo-planar imaging (EPI) sequences in one or two orthogonal planes, preferably frontal and sagittal [34]. In view of these guidelines, we propose the following 3-T MRI protocol. The fetal MRI examination is generally performed on pregnant women in the supine position, using a 3-T unit. Typically, one or two body matrix coils are used in combination with the spine array coil.

Our protocol includes T1-weighted three-dimensional (3D) GRE in the axial plane (repetition time [TR]/echo time [TE] = 6/1.3 ms; bandwidth 1,085 Hz/pixel; matrix size 156 × 256; slice thickness 3 mm) with and without fat suppression and T2-weighted FSE in axial, sagittal, coronal planes (TR/TE = 1,500/150 ms; bandwidth 592 Hz/pixel; matrix size 240 × 352; slice thickness 3 mm). The IVIM protocol includes a DWI EPI sequence on the transversal plane of the fetal body (TR/TE = 4,300 ms/54 ms, bandwidth 2,437 Hz/pixel, matrix size 92 × 114, 20 slices, slice thickness 3.5 mm, in-plane resolution 2.0 × 2.0 mm^2^. Diffusion encoding gradients are applied along three no-coplanar directions using 10 different *b*-values (0, 10, 30, 50, 70, 100, 200, 400, 700, 1,000 s/mm^2^). The total acquisition time of the IVIM DWI sequence is about 4 min (Table 3).

**Table 3:** Fetal 3-T magnetic resonance imaging protocol

Sequence	TA (min:s)	FOV (cm^2)	TR (ms)	TE (ms)	Flip angle (°)	Voxel size (mm^3^)	Number of slices	Acceleration (GRAPPA)	Other
T1 3D GRE fat saturated	0:19	24 × 30	6	1.3	9	1.2 × 1.2 × 3	30	3	Saturation method Dixon, breath-holding
T2 SS-FSE	0:55	27 × 30	1,500	152	135	0.8 × 0.8 × 2.5	25	2	Parallel saturation bands, free breathing
bSSFP	0:22	30 × 38	500	1.9	46	1.1 × 1.1 × 3	50	2	Offset frequency after scout evaluation, free breathing
Cine bSSFP	0:14	14 × 18	606	2.1	47	1.2 × 1.2 × 3	1	2	Real time, 20 phases, temporal resolution 1.5 phases/s, free breathing
IVIM DWI	2:50	38 × 30	4,500	67	−	1.7 × 1.7 × 3.5	20	2	*b*-values: 0, 10, 30, 50, 70, 100, 200, 400, 700, 1,000 s/mm^2^. Averages: 2, 2, 2, 2, 2, 3, 3, 4, 6, 9. Diffusion mode: 3D diagonal

Example protocol conducted on a 3-T scanner (MAGNETOM Vida, Siemens Healthcare, Erlangen, Germany). Examination is performed using the flexible body array combined with the spine array. *bSSFP* Balanced steady-state-free precession, *FOV* Field of view, *IVIM DWI* Intravoxel incoherent motion diffusion-weighted imaging, *T1 3D GRE* T1-weighted three-dimensional gradient-echo; *T2 SS-FSE* T2-weighted single-shot fast spin-echo, *TA* Acquisition time, *TE* Echo time, *TR* Repetition time

### T1-weighted 3D GRE

**Fig. 2:**
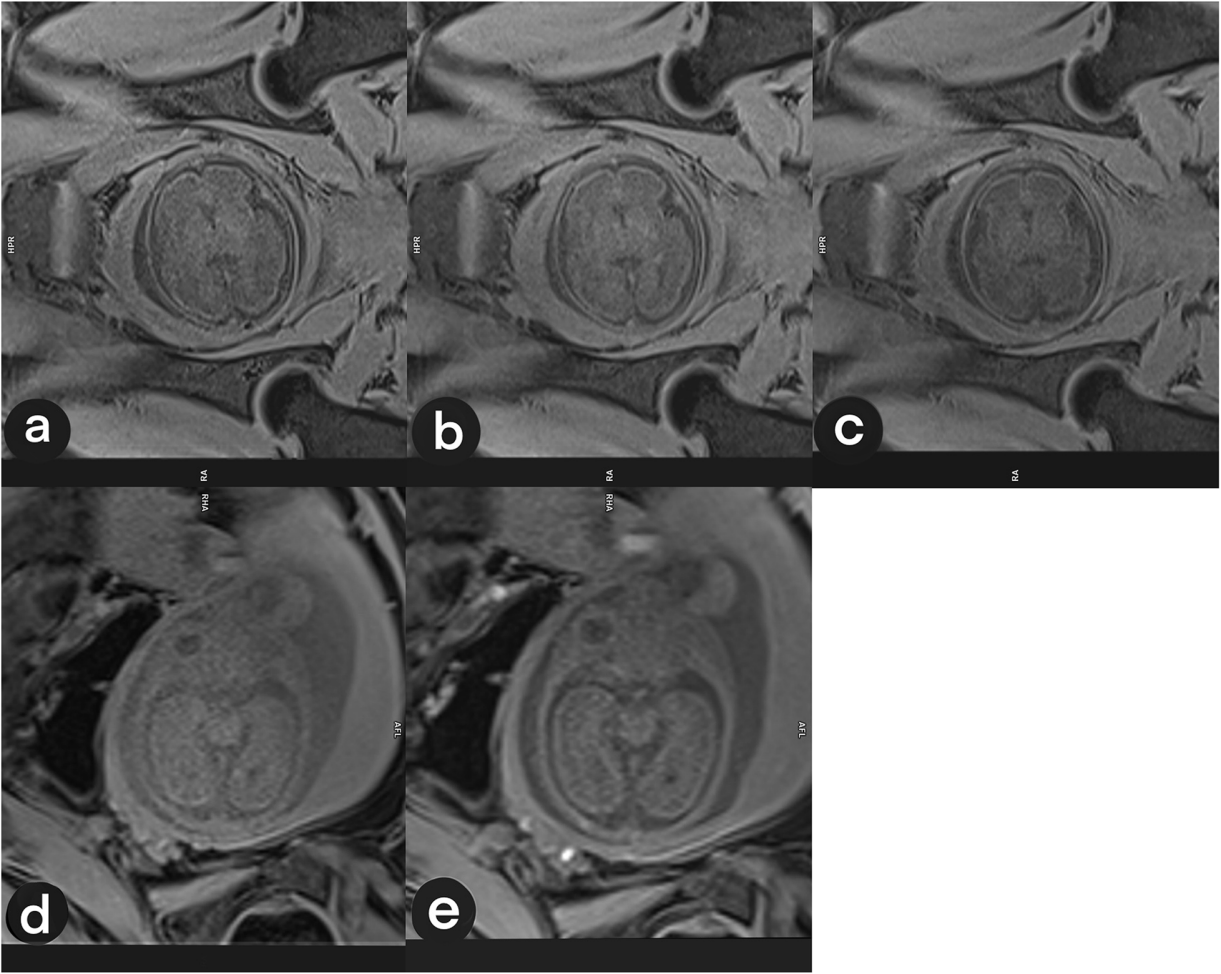
Transverse view of two fetal brains in different gestational ages, acquired by varying flip angle (FA) and repetition time (TR) with a T1-weighted 3D GRE fat-saturated sequence. **a** Repetition time = 4.1 ms and flip angle = 9°. **b** Repetition time = 6 ms and flip angle = 9°. **c** Repetition time = 7 ms and flip angle = 14°. **d** Repetition time = 4 ms and flip angle = 4°. **e** Repetition time = 6 ms and flip angle = 9°. A 29-week fetus is shown in the first row (**a**–**c**) and a 22-week fetus in the second row (**d**, **e**)

T1 contrast is generally decreased at 3 T with respect to 1.5 T [31]. The loss of T1 contrast may be compensated by a longer TR, leading to an increased acquisition time. The longer duration needed to create T1 contrast makes the sequence susceptible to fetal motion artifact. Parallel imaging combined with a T1-weighted 3D GRE sequence allows these acquisitions to be performed during breath-holding and hence can avoid some of the fetal motion. In Fig. 2, we show how T1 contrast is optimized at 3 T in different gas by modifying TR, TE, and flip angle in order to achieve the desired contrast. The sequence is a fat-saturated T1 3D GRE Dixon, with a field of view of 24 × 30 cm and 3-mm slice thickness [41].

### Single-shot fast spin-echo

**Fig. 3:**
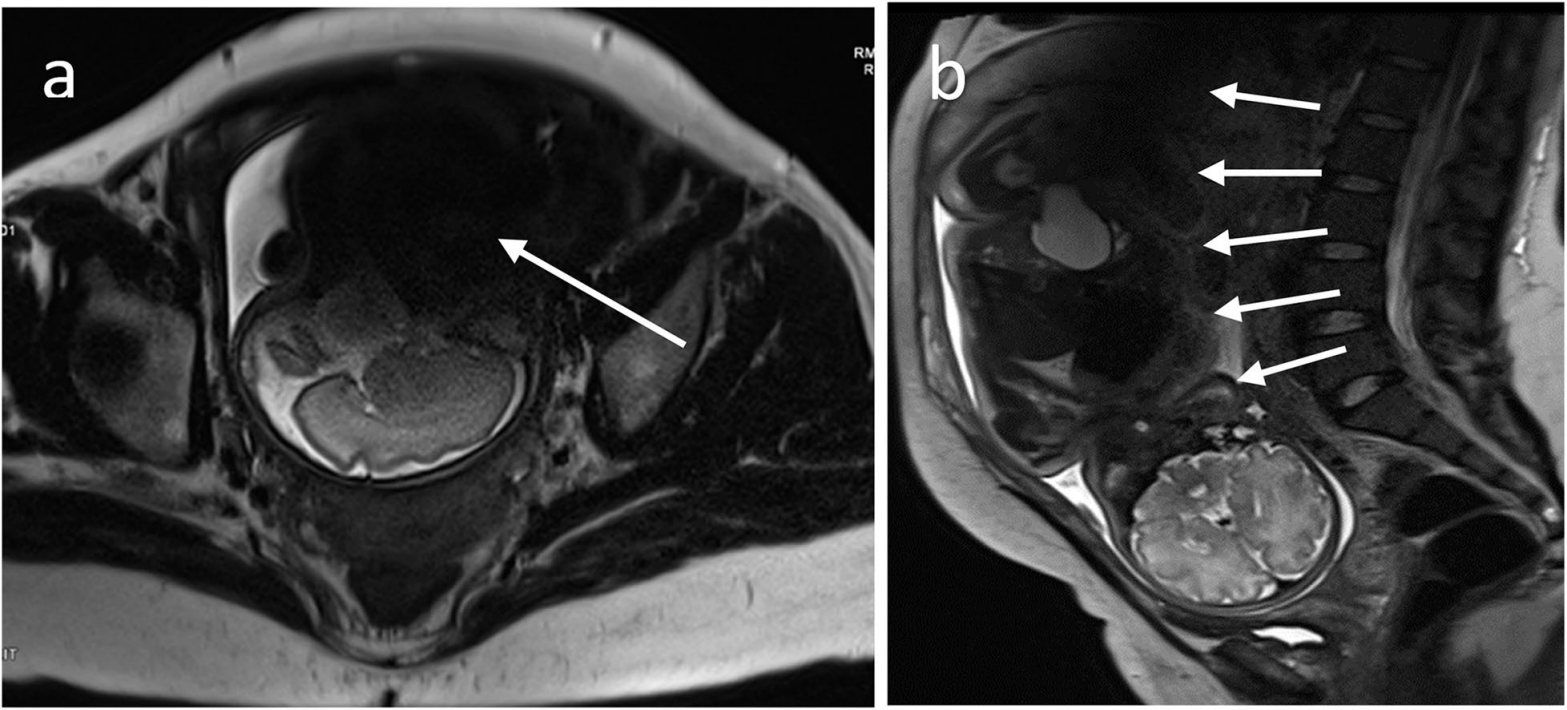
Sagittal and coronal views of a 34-week fetus acquired with a T2-weighted single-shot fast spin-echo sequence: sagittal plane of fetal brain corrupted by unpredicted fetal motion (arrow) (**a**). Coronal view of a fetal body corrupted by dielectric artifact (arrows) (**b**)

A free-breathing T2-weighted single-shot fast spin-echo (SS-FSE) sequence is often the technique of choice for fetal imaging [31]. This technique allows collection of an entire volume by freezing a single slice at a time. However, the images can be altered by unexpected fetal motion, and sometimes, it could be necessary to reacquire some images. One example of an image corrupted by fetal motion is shown in Fig. 3a. Another limiting factor of T2-weighted imaging in the fetus at 3 T is represented by the dielectric artifact, also known as *B*_1_ inhomogeneity [32]. The dielectric artifact strongly depends on the body region and the patient physiology [33], which makes it unpredictable.

**Fig. 4:**
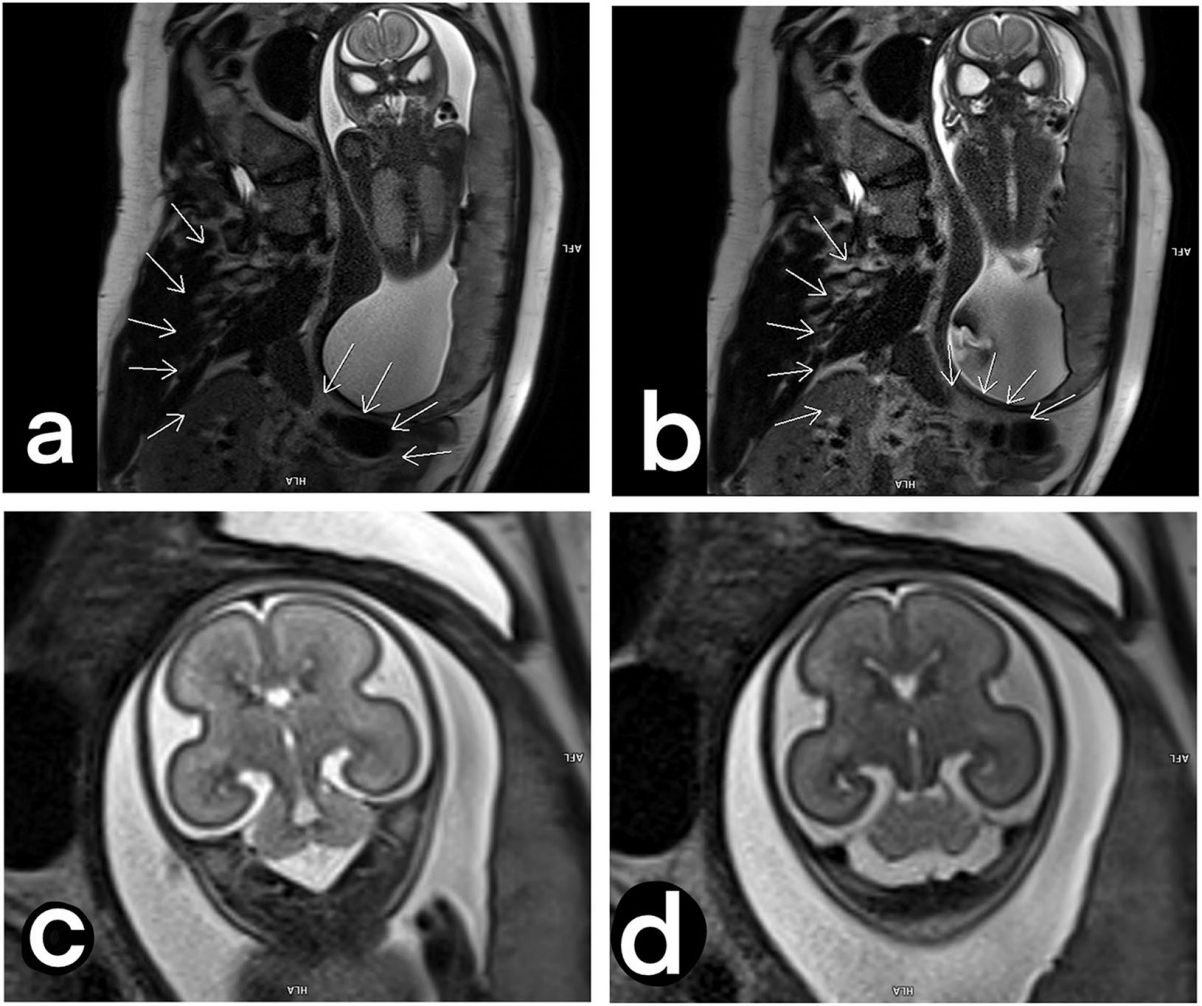
Coronal views of a fetal body before and after the setting of a prescan-B_1_ filter for T2-weighted single-shot fast spin-echo acquisitions, to describe dielectric artifacts. The signal intensity is spread to the image after the B1 filter was applied, showing up details around the mother’s organs (**a**, **b**). Images focused on the fetal brain, to describe how the body part of interest could be affected by the B_1_ filter: no B_1_-filter applied (**c**). B_1_-filter applied (**d)**

**Fig. 5:**
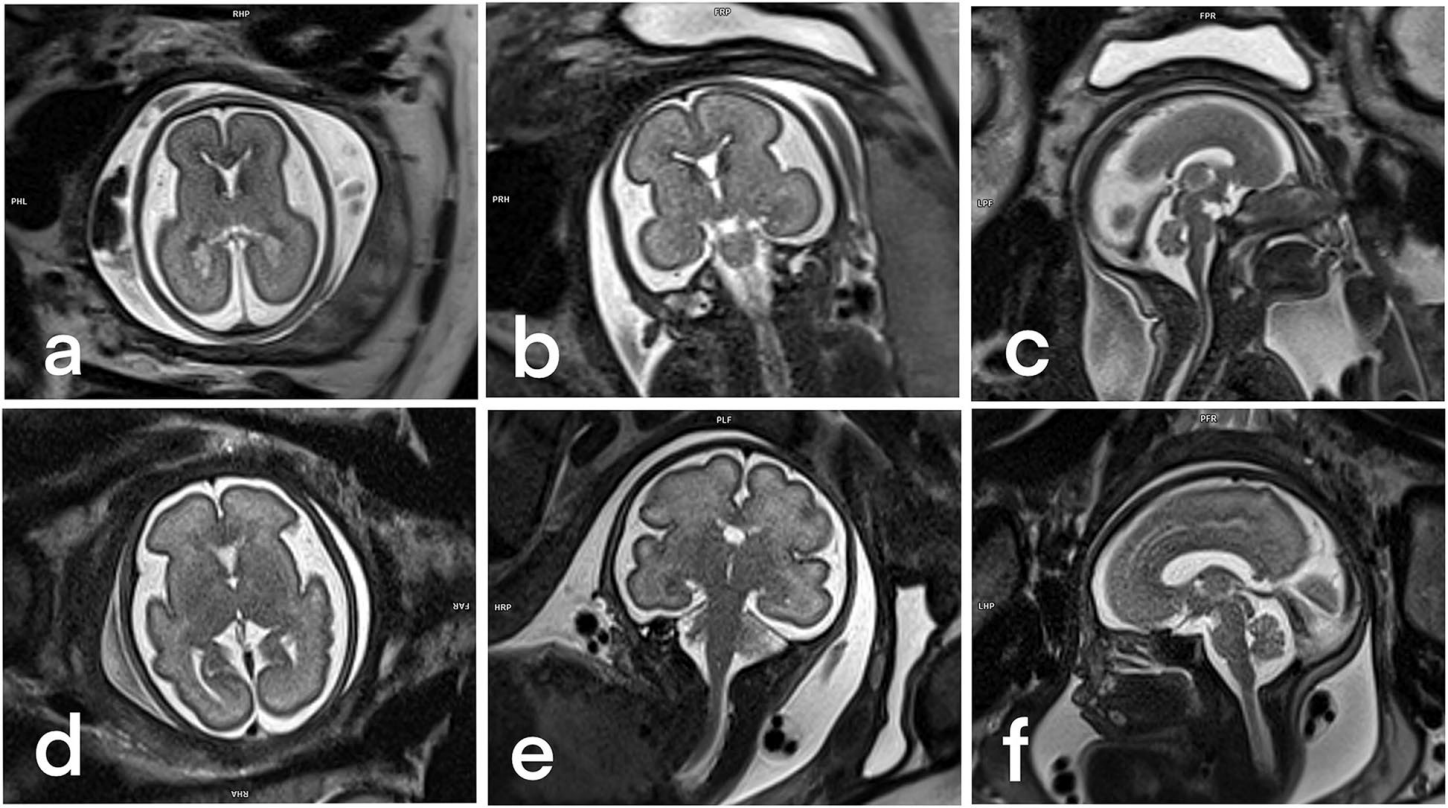
Transversal, coronal, and sagittal views of a T2-weighted single-shot fast spin-echo optimized sequence in fetuses of 22 weeks (**a**, **b**, **c**) and 28 weeks (**d**, **e**, **f**) 28 weeks

It is not unusual that the diameter of pregnant women exceeds 30 or also 50 cm, sizes that make the dielectric artifact hardly possible to avoid. Moreover, the large amount of amniotic fluid has detrimental effects on the B1 homogeneity within the field of view [32]. Using saturation bands and increasing the flip angle are good ways to attenuate the dark regions at the center of the image. However, these techniques can have the undesired side effect of increasing the deposited radiofrequency power, hence requiring a longer TR to remain within the SAR limits. Furthermore, a prescan-*B*_1_ filter can be applied, which is a homomorphous filter that can be used to reduce signal differences caused by dielectric resonances at field strengths of 3 T and higher and when the body coil is used [42]. The effect of the *B*_1_filter around the mother organs is shown in Fig. 4. Depending on the fetal position, it could be a good practice to move the shading artifact if the fetal brain is placed far from the body array coil. In Fig. 5, the axial, sagittal, and coronal views of the fetal brain in two different GA are illustrated.

### Balanced SSFP

**Fig. 6:**
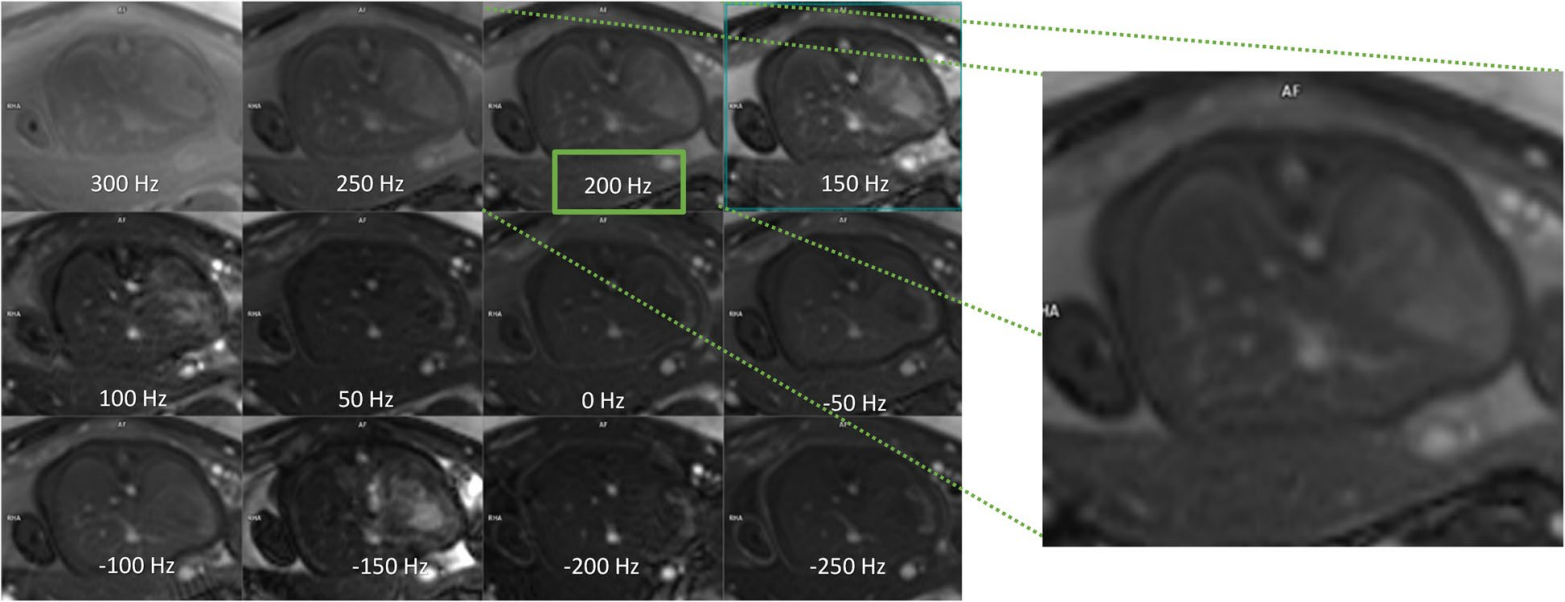
Single-slice transversal views of the “frequency scout” with twelve different offset frequencies. The frequency scout is planned in one or a few slices around the organ of interest. The “best” frequency is chosen by the operator and/or radiologist depending on the preferred contrast and/or the absence of the banding artifact around the point of interest, and the actual T2-weighted balanced steady-state-free precession is acquired

**Fig. 7:**
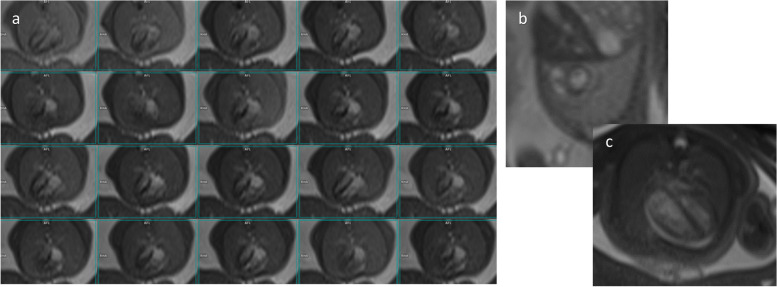
Single-slice cardiac views of a fetus' heart acquired with cine real-time bSSFP: 20-phases of 4-chamber view (**a**), short-axis view (**b**), selected 4-chamber view (**c**)

Balanced steady-state-free precession (bSSFP) sequences offer a very high signal-to-noise ratio and a T2/T1 image contrast. It is a type of GRE sequence in which the excited magnetization is maintained in the transverse plane using balanced gradient pulses [43]. It is useful for the evaluation of the heart and vessels because of the bright-blood signal, while in SS-FSE, the blood appears dark. To attenuate the increased banding artifacts, it may be necessary to adjust the offset frequency to move the banding artifacts outside the region of interest [44]. Due to the complex behavior of the signal evolution within different tissues, a different offset frequency also leads to different image contrasts. What was determined is that by modifying the frequency offset, also a different and maybe preferred contrast may be “chosen.” In Fig. 6, a single slice with a different offset frequency is acquired and then applied to the actual T2-weighted bright-blood acquisition. With bSSFP sequences, cardiac motion can be displayed in real time. It is best combined with triggering to ensure full coverage of the cardiac cycle. However, it can also be performed without the adoption of an external trigger. This makes real-time measurements an excellent alternative for examining uncooperative patients or patients who are unable to hold their breath [45]. The measurements are fast enough to avoid respiratory artifacts and can be achieved a temporal resolution of 1.5 phases/s. In Fig. 7, some examples of cardiac planes are shown.

## New perspectives, AI, and radiomics

New perspectives appear on the horizon for the application of fetal MRI (Table 4). 3D reconstructions of the fetal diaphragm can be used in cases of congenital diaphragmatic hernia to visualize, locate, classify, and quantify the defect. Thanks to the segmentation of the diaphragm and the diaphragmatic defect, made possible by the use of dedicated software, 3D visualization is made possible on three orthogonal planes. With these reconstructions, it is possible to optimize postnatal surgical planning, predict the need for patch placement, and customize the patch design based on 3D-printable templates [46].

**Table 4:** New approaches, AI, and radiomics fetal magnetic resonance imaging (MRI) studies

	**First author**	**Year**	**Title**	**Cases**	**Results**
1	Prayer F	2019	Three-dimensional reconstruction of defects in congenital diaphragmatic hernia: a fetal MRI study	46	Standardized *in vivo* fetal MRI allows reproducible extraction of lung radiomics features
2	Lloyd D. F. A	2019	Three-dimensional visualization of the fetal heart using prenatal MRI with motion-corrected slice-volume registration: a prospective, single-center cohort study	85	Fetal cardiovascular MRI, incorporating novel open-source 3D image processing algorithms, can significantly improve the visualization of major vascular abnormalities in late-gestation fetuses compared with two-dimensional MRI
3	Kulseng C. P. S	2023	Automatic placental and fetal volume estimation by a convolutional neural network	193	The correctness of neural network volume estimation is comparable to human performance; the efficiency is substantially improved
4	Shen L	2022	Attention-guided deep learning for gestational age prediction using fetal brain MRI	741	The proposed regression algorithm provides an automated machine-enabled tool with the potential to better characterize in utero neurodevelopment and guide real-time gestational age estimation after the first trimester
5	Singh A	2020	Deep predictive motion tracking in magnetic resonance imaging: application to fetal imaging	N/A	The proposed real-time deep predictive motion tracking technique can be used to assess fetal movements, to guide slice acquisitions, and to build navigation systems for fetal MRI
6	Xu J	2019	Fetal pose estimation in volumetric MRI using a 3D convolution neural network	N/A	The proposed method achieves mean error of 4.47 mm (~ 1.5 pixels) and a percentage of correct detection of 96.4%, which indicates that deep neural networks are able to identify key features for fetal pose estimation from time frames in low-resolution, volumetric echo-planar imaging data from pregnant mothers. Furthermore, the total processing time of the proposed framework is less than 1 s, potentially enabling low latency tracking of fetal pose in fetal MRI
7	Prayer F	2023	Fetal MRI radiomics: noninvasive and reproducible quantification of human lung maturity	30	Standardized *in vivo* fetal MRI allows reproducible extraction of lung radiomics features
8	Song F	2023	Predicting the risk of fetal growth restriction by radiomics analysis of the placenta on T2WI: a retrospective case–control study	202	MRI-based placental radiomics could accurately predict fetal grow restriction. Moreover, combining placental MRI-based radiomic features with ultrasound indicators of the fetus could improve the diagnostic accuracy of fetal grow restriction

In recent years, the use of AI software through prerocessing and postprocessing MRI techniques is proposing itself as a tool for automating the acquisition of images, improving quality, and time-saving [47].

AI allows to speed up the time-consuming process of segmentation of different organs through automation. Kulseng et al. [48] trained a convolutional neural network to automatically estimate placental and fetal volumes and subsequently compared the values calculated by the AI with those calculated manually. The values obtained by AI were strongly in agreement with those calculated manually both as regards the placental volumes and as regards the fetal volumes, demonstrating how the use of AI is advantageous, reducing the duration of the process from more than 1 h to a few seconds.

AI can be leveraged to estimate GA. Shen et al. [49] built an attention-guided-multi view deep learning network, trained with a heterogeneous dataset of 741 magnetic resonance images of normally developed fetuses from 19 to 39 weeks of GA, capable of predicting GA with a mean absolute error of 6.7 days.

The uncontrollable, large, and irregular fetal movements make the MRI acquisition process highly operator dependent, increase the use of the magnet with the resulting costs, and determine long examination times often unbearable for the patients. To solve this limitation, the use of a new motion correction algorithm has been proposed by Singh et al. [50]. They developed a new real-time image-based motion tracking method based on deep learning that predicts fetal head motion directly from acquired images. The system exploits a deep regression recurrent neural network model consisting of two main parts: an encoder and a decoder. By concerting this technique with additional real-time components and implementing it in MRI, it would be possible to trace the movements of the fetus’ head every time the different sections are acquired and to provide support to the operator in orienting the scans. Similarly, Xu et al. [51], with the aim of reducing the examination times, have described a deep learning algorithm capable of predicting the position of the fetus using 15 key points, capable of tracing the fetus, positioned in different body parts (ankles, knees, hips, bladder, shoulders, elbows, wrists, and eyes). Other authors [52] showed how using an open-source reconstruction algorithm with motion correction software on 2D images is possible to obtain high-resolution 3D images of the fetal thorax and vascular structures. The 3D images obtained show good spatial agreement with ultrasound measurements and significantly improved visualization and diagnostic quality compared to the source 2D MRI data.

Finally, among the new frontiers of fetal MRI, we find radiomics. By radiomics, we mean an image analysis process where a large number of data are extrapolated from the image data using predefined statistical operations with the aim of obtaining otherwise visually imperceptible features that characterize a specific tissue or predict a certain outcome [53]. Prayer et al. [54], studying the 1.5-T MRI data of 30 fetuses, demonstrated that in using a standardized fetal MRI protocol, it is possible to extract reproducible lung radiomics features that can be useful to complete the observed-to-expected lung volume in predicting neonatal outcome. Song et al. [55] demonstrated that MRI-based placental radiomics can accurately predict FGR, and that by combining the radiomic features obtained with ultrasound indicators of the fetus, it is possible to improve the diagnostic accuracy of FGR.

## Conclusions

Fetal MRI represents a second-line investigation to evaluate fetal malformations. In the last decade, new field of interests have been investigated and developed with the aim to go beyond the morphological data and a qualitative assessment. The aim was also to improve diagnosis (reducing diagnostic error), classify cases into risk classes, and select new parameters and measurements in the era of quantitative imaging and precision medicine.

The role of DWI is now widely recognized for all the additional information on fetal malformation. The introduction of 3-T magnets improved the contrast and spatial resolution of fetal MRI, even if movement artifacts may reduce the quality of the images particularly in fetuses in early period of gestation. As regards IVIM, some limitations need to be overcome to make it suitable for clinical application. The main one is the duration of the experiment, as it involves the acquisition of DWI at different *b*-values (at least 10). A long duration involves a greater risk of fetal movement and too long diagnostic times. This drawback could be overcome by using parallel imaging acquisitions and motion correction techniques.

AI and the development of reconstruction algorithm with motion correction software on 2D images can contribute to obtain high-resolution 3D images of fetal brain, thorax, and vascular structures and will represent a new frontier in fetal MRI. Moreover, with the applications of AI in the segmentation process and in the estimation of GA and with the advent of radiomics, new horizons are opening up.

## Data Availability

Not applicable.

## Abbreviations

2D: Two-dimensional
3D: Three-dimensional
ADC: Apparent diffusion coefficient
AI: Artificial intelligence
bSSFP: Balanced steady-state-free precession
CAIPI: Controlled aliasing in parallel imaging
DWI: Diffusion-weighted imaging
EPI: Echo-planar imaging
FGR: Fetal growth restriction
FLAIR: Fluid-attenuated inversion recovery
GA: Gestational age
GRE: Gradient echo
IVIM: Intravoxel incoherent motion
QSM: Quantitative susceptibility mapping
SAR: Specific absorption rate
SGA: Small for gestational age
SS-FSE: Single-shot fast spin-echo
SWI: Susceptibility-weighted imaging
US: Ultrasonography
